# Effects of citral on oxidative stress and hepatic key enzymes of glucose metabolism in streptozotocin/high-fat-diet induced diabetic dyslipidemic rats

**DOI:** 10.22038/ijbms.2018.26889.6574

**Published:** 2019-01

**Authors:** Chetna Mishra, Monowar Alam Khalid, Nazmin Fatima, Babita Singh, Dinesh Tripathi, Mohammad Waseem, Abbas Ali Mahdi

**Affiliations:** 1 Department of Biochemistry, King George’s Medical University, Lucknow-226003, Uttar Pradesh, India; 2 Department of Environmental Science, Integral University, Lucknow-226021, Uttar Pradesh, India; 3 Department of Physiology, King George’s Medical University, Lucknow- 226003, Uttar Pradesh, India

**Keywords:** Carbohydrate metabolism, Citral, Diabetes, Dyslipidemia, Enzymes, Oxidative stress, Streptozotocin

## Abstract

**Objective(s):**

Phytochemicals such as polyphenols, alkaloids, and terpenoids, protect against the development of early stages and complications of diabetes mellitus according to various reports. The aim of this study was to measure the anti-dyslipidemic and anti-diabetic effects of Citral on high-fat-diet (HFD) and streptozotocin (STZ) induced diabetic dyslipidemic rats and to see also its effect on carbohydrate metabolic regulatory enzymes in the liver.

**Materials and Methods:**

Rats were kept on a high-fat diet for 2 weeks, then diabetes was induced by a single dose of STZ (35 mg/kg/BW, intraperitoneally), Citral was administered orally at a dose of 45 mg/kg/BW for 28 days to diabetic rats. Blood glucose, plasma insulin, and lipid profile in blood were studied. Antioxidant activities were assayed in the liver, pancreas, and adipose tissues. Carbohydrate metabolic enzymes of the liver were also studied in diabetic dyslipidemic rats.

**Results:**

The results of this study confirmed that administration of Citral significantly (*P*<0.05) decreased the blood glucose level and increased plasma insulin in diabetic rats. Citral also improved oxidative markers along with anti-oxidative enzymes of the liver, adipose tissue, and pancreas in the HFD/STZ group. Citral also regulated the activity of the glucose-metabolic enzymes in the liver. The results of the present study were compared to Glibenclamide, which is a standard oral drug for lowering the blood sugar.

**Conclusion:**

Results may show that Citral possesses anti-dyslipidemic activity as well as anti-diabetic activity and also regulates the enzyme activity of glycolytic and gluconeogenic processes in the liver.

## Introduction

Diabetes mellitus or type 2 diabetes is a worldwide ailment affecting millions of people. According to the WHO report, more than 420 million adults were living with diabetes at the global level in 2014. India is the second leading country affected by diabetes after China. According to WHO’s 2015 data, more than 69.2 million people are estimated to have diabetes in India (1, 2). Diabetes is a metabolic disturbance generally diagnosed by high blood glucose (hyperglycemia) resulting from insulin deficiency or insulin resistance. In diabetes, hyperglycemia produces free radicals or reactive oxygen species which leads to lipid peroxidation, insulin resistance, tissue injury, and other problems like retinopathy, cardiopathy, neuropathy, and nephropathy, etc. (3–5). The increase of lipid peroxidation and insulin resistance by abnormalities in lipid levels are associated with dyslipidemia or hyperlipidemia (6, 7).

In addition to hyperglycemia, disturbance in the metabolism of lipids, carbohydrates, and proteins may also occur in diabetes (8). Any interruption in the pathway of metabolism of glucose and lipids may cause glucotoxicity and lipotoxicity in diabetes which leads to impaired beta-cell functions, and increases insulin resistance in muscles, liver, and adipose tissue (9, 10). In type 2 diabetes, insulin insensitivity and insufficiency lead to a decrease in consumption of blood glucose by the liver, muscles, and adipose tissue thus, impaired insulin action directly affects the glucose production in the liver (11). Glucose homeostasis, including gluconeogenesis and glycolysis process, involves the regulation coordination of several metabolic pathways, which is caused by poor carbohydrate use as a result of insulin deficiency (12). 

A new therapeutic approach is needed for the treatment of high blood glucose level, dyslipidemia, and oxidative stress simultaneously because all three are responsible for other complications in the diabetic patient. Therefore, herbal plant anti-diabetic drugs with anti-dyslipidemic property and antioxidant potential could be the best option for treatment of the patients with no side effects. In addition, studying the effects of phytochemicals on glucose metabolism is an interesting area to explore.

*Cymbopogon* *citratus*, Stapf (Lemongrass) is an herb widely used in tropical countries as a folk medicine in various diseases (13). Antibacterial, anti-diarrheal and antioxidant activities have also been reported in the plant. It is believed that flavonoids, phenolic compounds, terpenoids, etc. are phytocompounds present in plants and can be responsible for various biological activities. A major component of *C.* *citratus* is Citral, which is a mixture of two terpenoids (geranial and neral) with the molecular formula C_10_H_16_O (14). Thus, the present work is designed to explore the antidiabetic, antidyslipidemic, and antioxidant effects of Citral, isolated from *C. citratus*, in high-fat diet and streptozotocin-induced diabetic dyslipidemic rats. We are also investigating the effect of Citral on glucose metabolic enzymes of the liver.

## Materials and Methods

***Chemicals and reagents***

 All chemicals used in this experimental study were of analytic grade and were purchased from Sigma Aldrich, Chemicals Pvt. Ltd USA. 

***Plant material***

*Cymbopogon citratus *(DC) Stapf, belonging to the family Poaceae, holds a reputed position in both Ayurvedic and Unani systems of medicine. The *C. citratus *whole plant was procured from National Botanical Research Institute (NBRI) Lucknow.

***Ethanolic plant extract preparation***

*C. citratus* leaves were powdered in a blender after drying in the shade. After that 100 ml of 50% ethanol was macerated with 20 g powder content for three days, this process was repeated four times. Then the plant extract was filtered through Whatman filter paper and concentrated by using rota evaporator (Buchi Rotavapour R-114) to obtain a dry powder. We found 6.32% of the total yield of the extract.

***RP-HPLC analysis of plant extract***

Liang *et al*. 2004 method with some modification was used to isolate Citral by Reverse Phase-High Performance Liquid Chromatography (RP-HPLC) from 50% ethanolic extract of *C. citratus *(15). An Agilent 1260 infinity quaternary LC series system consisting of 1260 infinity diode array detector (DAD) and quaternary solvent delivery system with thermostatted autosampler, equipped with an Agilent zorbax C-18 column (4.6 mm–250 mm; 5 um) was used in this study. A stock solution of citral (1 mg/ml) was prepared in ethanol and was further diluted to obtain 25 ng/ml, 50 ng/ml, and 100 ng/ml concentrations of the standards. For the sample preparation, plant extract was dissolved in ethanol to get a final concentration of 5 mg/ml. Acetonitrile: deionized water with 0.01% phosphoric acid; (0.45:0.55) was used as a mobile phase for the separation of Citral. The flow rate was 1 ml/min and the detection wavelength was at 254 nm.

***Animals***

Male albino Sprague–Dawley rats, weighing 200–250 gm were taken in this experiment. All rats were kept at a temperature of 20 °C in the Central Animal house of King George’s Medical College. They had standard diet-pellets (Ashirvad Industries, Chandigarh) and water* ad libitum*. All experiments were performed as per the directives of the institutional Animal Ethics Committee (40/IAEC/2013).

***Experimental design and induction of type 2 diabetes***

In this study, we induced diabetes mellitus by administration of a small dose of Streptozotocin (STZ) in high-fat diet (HFD) fed rats according to Srinivasan *et al. *methodology (16). Rats were fed High-fat diet for 2 weeks. After 2 weeks, an injection of a single dose of STZ (35 mg /kg) dissolved in 50 m M cold citrate buffer (pH 4.5) was given intraperitoneally (IP). The feeding with HFD was continued till the end of the experiment. On the third day of STZ injection, blood glucose above 180 mg/dl confirmed diabetes in rats. 

***Treatment schedule***

Citral, as well as fenofibrate and glibenclamide, were macerated in triple distilled water containing 2% gum acacia and fed orally once daily for 14 days by gastric tube at the doses mentioned below. The dose of citral was decided according to the literature (17). Glibenclamide and fenofibrate are used as standard drugs for diabetes and hyperlipidemia, respectively (18, 19). The study comprised the following groups:

Group-1: Normal healthy rats received saline (control)

Group-2: HFD/STZ treated rats received saline (diabetic control)

Group-3: HFD/STZ rats received citral (30 mg/kg bw/day) once a day

Group-4: HFD/STZ rats received glibenclamide (600 µg/ kg BW) once a day

Group-5: HFD/STZ rats received fenofibrate (500 mg/kg BW) once a day

***Tissue preparation***

On the last day of the experiment, rats were anesthetized by ether and blood was drawn from the dorsal aorta for the estimation of blood glucose, insulin, and lipid profile. After that rats were sacrificed and their liver, adipose tissues, and pancreas were taken out immediately and washed with ice-cold saline. All the tissues were homogenized with 0.1 M phosphate buffer (pH 7.4) and then centrifuged for 5 min at 4 °C. The supernatant was collected and used for various biochemical assays.

***Assessment of oral glucose tolerance test (OGTT)***

A day before the end of the experiment all rats of the five groups were kept fasting. Next morning, blood samples were withdrawn from the tail vein of overnight fasting rats and blood glucose was measured at 0 30, 60, 90, and 120 min interval after the oral administration of glucose solution (3 g/kg BW)to all rats (20).

***Assessment of diabetic and dyslipidemic parameters***

The level of glucose in blood was measured by using the method of Trinder (21). Plasma insulin was measured by rat insulin enzyme-linked immunosorbent assay (ELISA) kit (22). Glycosylated hemoglobin (HbA1c) was estimated according to the method of Goldstein *et al* (23). Total Protein was estimated by a known method (24). Low-density lipoprotein (LDL-c) was measured by following a method known in literature (25). Free fatty acids (FFAs) were estimated spectrophotometrically by a known method (26). Measurement of Total Cholesterol (TC) was done by spectrophotometrically by another method (27). Phospholipids (PLs) were measured by following a method known in literature (28) and triglycerides (TGs) were measured according to the literature (29).

***Assessment of oxidative-antioxidative status in the liver, adipose tissue, and pancreas***

Malondialdehyde (MDA) level in Lipid peroxidation was estimated according to a known method (30) in homogenates of the liver, pancreas, and adipose tissues. Protein carbonyl (PC) groups were measured by a spectrophotometric method with the use of the carbonyl specific reagent dinitrophenylhydrazine (DNPH). The optical density of which was read in UV range at 280 nm on a spectrophotometer (31). Superoxide dismutase (SOD) activity was assayed using a known method with the absorbance read at 560 nm against the blank (32). Catalase (CAT) activity was determined by following the method described in literature (33). Glutathione peroxidase (GPx) activity was measured by a known method. Enzyme unit was defined as nmol of NADPH oxidized /min/mg protein (34). Glutathione reductase (GR) was estimated by following another method. Results were expressed as unit/min/mg protein (35). Reduced glutathione (GSH) content level was estimated by the method of Ellman in all taken tissues (36).

***Assessment of carbohydrate metabolism enzymes in the liver***

Glucokinase (GK) activity was assayed by a method from the literature. The enzyme units are expressed in min/mg protein (37). Hexokinase was estimated by following another known method. Optical density was recorded after 30 sec intervals at 340 nm (38). Glucose-6-phosphatase (G6P) activity was estimated according to the method described by Fiske (1925). The enzyme units are expressed as µmol Pi release /min/mg protein (39). Pyruvate kinase (PK) was assayed according to a known method (40). Lactate dehydrogenase (LDH) is a cytoplasmic enzyme, measured by following the method of Decker *et al* (41).

***Statistical analysis***

Results were expressed as mean±SE and were analyzed on Graph Pad Prism 5 software using Student’s t-test and one-way ANOVA (analysis of variance). *P<0.05, **P<0.01, ***P<0.001 were used as the criterioa for significance. 

## Results

***Compound isolation and validation using RP-HPLC***

**Figure 1:**
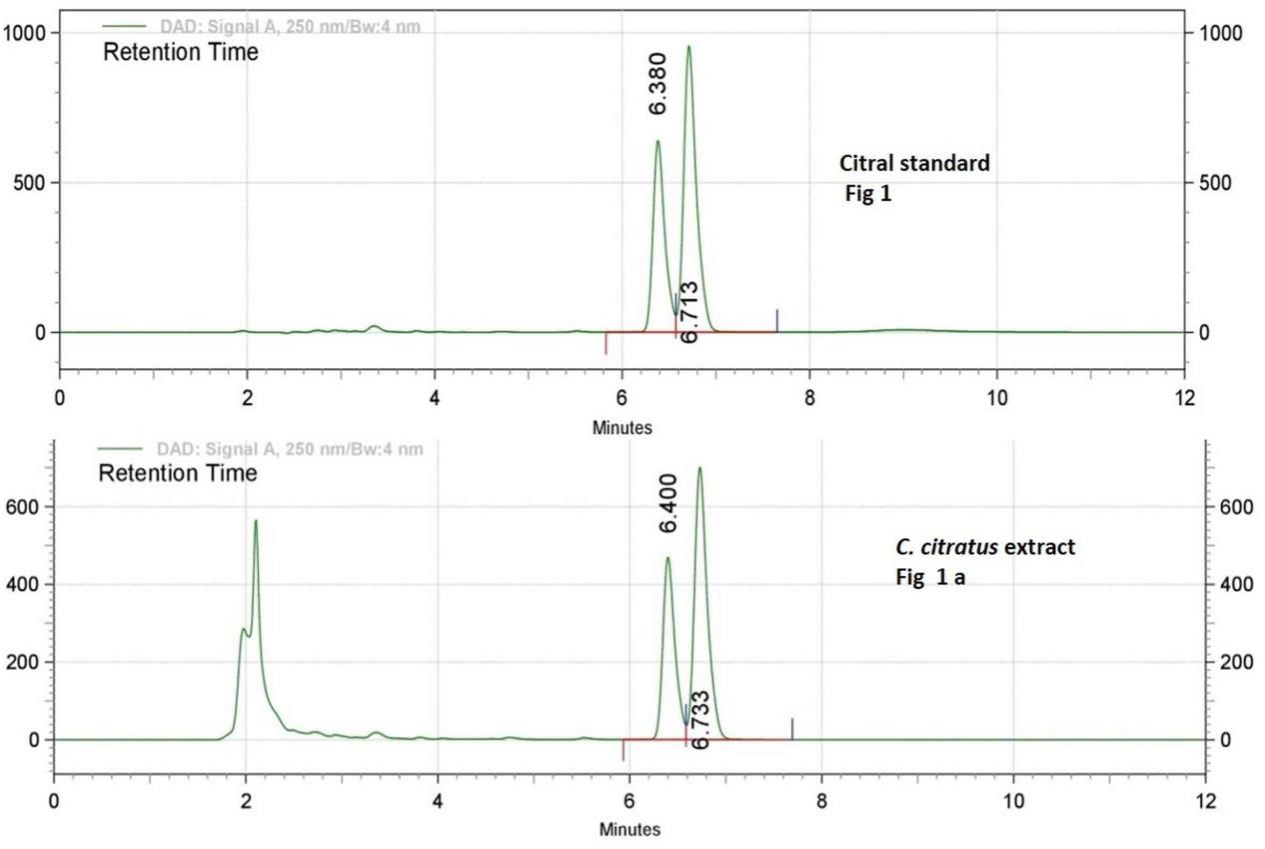
First chromatograms of lyophilized 50% ethanolic extract of* Cymbopogon citratus* leaves (5 mg/ml) with peak retention time (Rt) of Neral or Citral 1=6.44 min and Geranial or Citral 2=6.73 min. Second chromatogram of Citral standard showing two peaks of Citral 1 or neral; RT=6.38 and Citral 2 or geranial; RT=6.71

Reverse phase-high performance liquid chromatography (RP-HPLC) analysis was done for standardization of plant extract. Commercially available Citral was used as a standard, which is a mixture of two terpenoids, Neral and Geranial. Detection and isolation of Citral from the *C. citratus* extract was carried out by using a photodiode array detector at 254 nm wavelength. The retention time of Citral-1 (Neral) and Citral-2 (Geranial) are 6.33 and 6.76, respectively, and the peaks of Citral-1 and -2 were collected from the plant extract sample. RP-HPLC chromatogram of standard and *C. citratus* extract is shown in Figure 1, which confirms the presence of Citral in the extract sample. These results authenticate the standardization and validation of extract. 

***Effect of citral on body weight and OGT test***

Table 1 shows the body weight of control and experimental groups of rats, which were checked up to 30 days. The body weight of diabetic dyslipidemic rats was significantly decreased as compared to normal rats. In the initial days of experiments there was no significant difference found in body weight of Citral administered group, but after fifteen days the body weight was significantly increased in Citral, glibenclamide, and fenofibrate-treated rat groups when compared to the diabetic rats group. Results of the oral glucose tolerance test of all the groups at different time points (0, 30, 60, 90, and 120 mins) have been summarized in Table 2. 

***Effect of citral on levels of blood glucose, insulin, and HbA1c***

Table 3 represents the data of blood glucose concentration, insulin level, and HbA1c of all five groups. It has been observed that blood glucose and HbA1c levels have significantly decreased in citral treated rats along with the significantly increased level of insulin as similar to glibenclamide treatment when compared to the diabetes control group.

***Effect of citral on lipid profiles***

The data of lipid profile have been reported in Table 4. In which the diabetic control group showed a significant (*P*<0.05) increment in serum TC, TG, FFAs, LDL-C, and phospholipids levels compared to the control group. Treatment of the citral group significantly (*P*<0.05) restored all the changes in lipid profile and FFAs compared to the HFD/STZ group.

***Effect of citral on oxidative stress***

**Figure 2:**
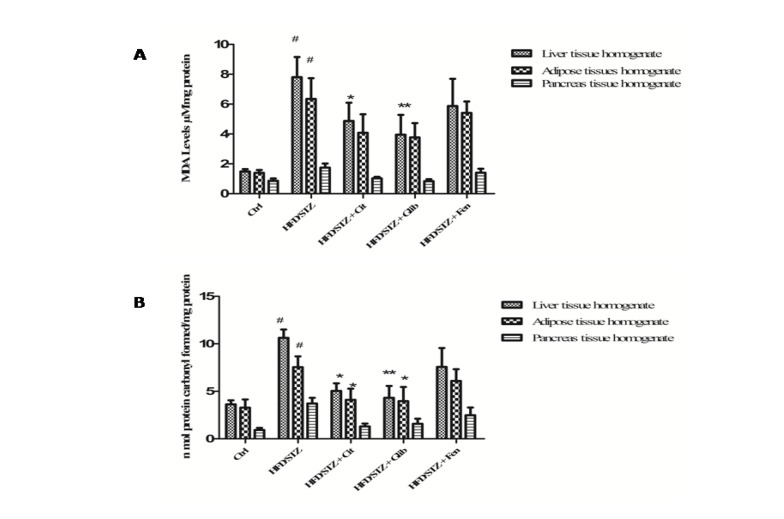
(a) Effect of citral (30 mg/kg bw) supplementation on MDA level of STZ + HFD induced diabetic dyslipidemic rats. (b) Effect of citral (30 mg/kg/BW) supplementation on protein carbonyl content of STZ + HFD induced diabetic dyslipidemic rats. Ctrl: Control rats; HFD/STZ: Diabetic rats; HFD/STZ + Cit: Diabetic rats treated with citral; HFD/STZ + Glib: Diabetic rats treated with standard drug glybenclamide; HFD/STZ + Fen: Diabetic rats treated with standard drug fenofibrate. # denotes significant difference compared with control rats; * *P*<0.05; ** *P*<0.01;*** *P*<0.001 denotes significant difference compared with diabetic control

Effect of citral on MDA level was measured in the liver, pancreas, and adipose tissues of all groups in this study. It has been found that MDA level was significantly (*P*<0.05) increased in the homogenates of liver (7.81±1.35 µM/mg), adipose (6.34±1.40 µM/mg), and pancreas (1.74±0.27 µM/mg protein) of diabetic group rats as compared to liver, adipose, and pancreas tissues (1.49±0.15, 1.39±0.18 and 0.86±0.16 µM/mg, respectively) of normal control rats. However, a significant reduction in the level of MDA was observed in the liver homogenate tissue (4.87±1.23 µM/mg) of Citral treated rats as well as glibenclamide (3.95±1.32 µM/mg) treated rats but the statistically insignificant recession was observed in other tissues of the citral group when compared to diabetic control rats (Figure 2a). 

Citral supplementation significantly (*P*<0.01) attenuated protein carbonyl content in the liver (5.04±0.8 nmol/mg) and adipose tissue homogenates (4.11±1.14 nmol/mg) as compared with the liver and adipose tissues homogenates (10.62±0.87 and 7.54±1.17 nmol/mg, respectively) of the diabetic control group (Figure 2b). 

**Figure 3:**
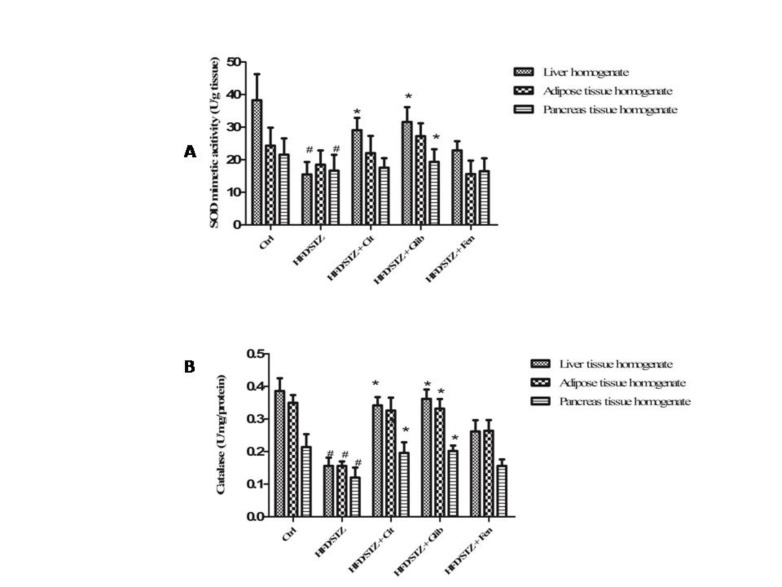
(a) Effect of citral (30 mg/kg BW) supplementation on SOD mimetic activity of STZ+HFD induced diabetic dyslipidemic rats. (b) Effect of Citral supplementation on Catalase activity of STZ+HFD induced diabetic dyslipidemic rats. Ctrl: Control rats; HFD/STZ: Diabetic rats; HFD/STZ+Cit: Diabetic rats treated with Citral; HFD/STZ+Glib: Diabetic rats treated with standard drug glybenclamide; HFD/STZ+Fen: Diabetic rats treated with standard drug fenofibrate. # denotes significant difference compared with control rats; * *P*<0.05; ** *P*<0.01;*** *P*<0.001 denotes significant difference compared with diabetic control

Significant decrease in SOD activity in liver homogenate (15.49±3.81 U/g tissue) and pancreas tissue homogenates (18.47±4.40) was found in the diabetic control group as compared to SOD activity in liver and pancreas homogenates (38.26±8.00 and 21.54±5.02 U/g tissue, respectively) of normal control rats. Citral has been found to significantly (*P*<0.05) improve the activity of SOD in liver tissues only (29.06±3.71 U/g tissue) (Figure 3a). 

**Figure 4:**
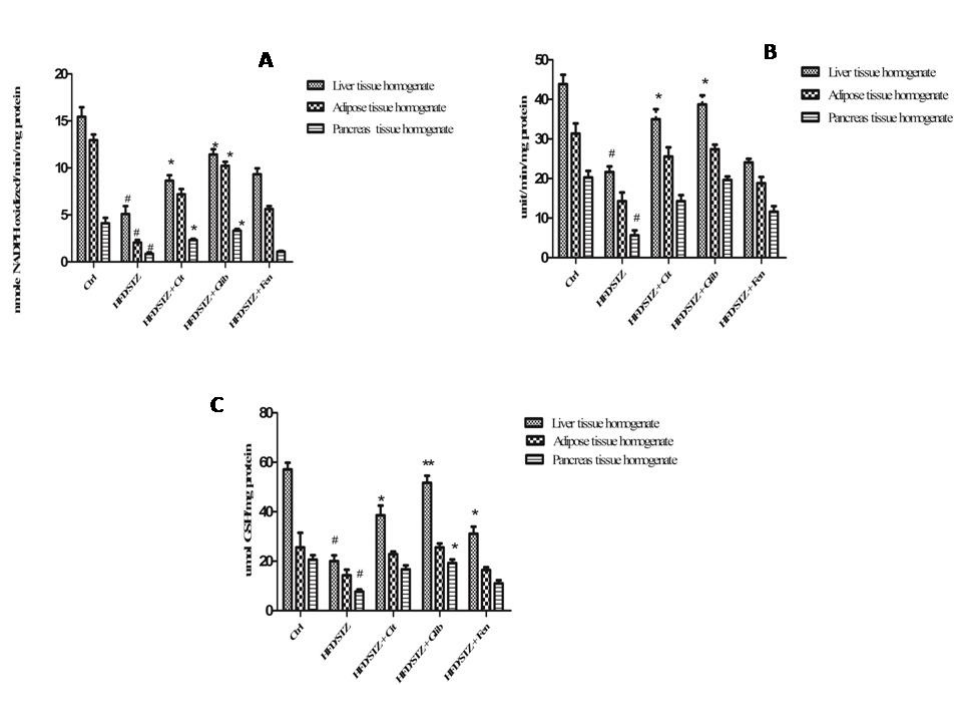
(a) Effect of citral (30 mg/kg BW) on GPx activity of STZ + HFD induced diabetic dyslipidemic rats. (b) Effect of citral supplementation on GR activity of STZ + HFD induced diabetic dyslipidemic rats. (c) Effect of citral supplementation on GSH activity of STZ+HFD induced diabetic dyslipidemic rats. Ctrl: Control rats; HFD/STZ: Diabetic rats; HFD/STZ+Cit: Diabetic rats treated with citral; HFD/STZ+Glib: Diabetic rats treated with standard drug glybenclamide; HFD/STZ+Fen: Diabetic rats treated standard drug fenofibrate. # denotes significant difference compared with control rats; * P<0.05; ** *P*<0.01;*** *P*<0.001 denotes significant difference compared with diabetic control

Significant decrease in catalase activity was observed in all three tissues: liver, adipose and pancreas homogenates of diabetic control rats (0.15±0.02, 0.16±0.01 and 0.12±0.03 U/mg/ protein, respectively) when compared with the same tissues of normal control rats (0.38±0.03, 0.35±0.02 and 0.21±0.04 U/mg/ protein, respectively). Citral significantly (*P*<0.05) restored the activity of Catalase in liver and pancreas tissue homogenates (0.34±0.02 and 0.19±0.03 U/mg/ protein, respectively) as compared to the diabetic control group (Figure 3b). Citral treatment has shown significant (*P*<0.05) increase in Gpx activity of the liver (8.63±0.588 nmol/min/mg) and pancreas tissues (2.32±0.12 nmol/min/mg) as compared to liver (5.09±0.84 nmol/min/mg and pancreas (0.86±0.14 nmol/min/mg) of diabetic control rats (Figure 4a). Whereas we observed GR activity and GSH level of Citral treated rats showed a significant increase in liver tissues only (34.97±2.53 unit/min/mg, 38.53±3.95 µmol/mg, respectively), compared with diabetic rats (21.64±1.34 unit/min/mg, 20.06±2.30 µmol/mg, respectively) (Figure 4b and 4c). 

***Effect of citral on carbohydrate metabolism enzymes of the liver***

Table 5, represents the activity of glycolytic and gluconeogenic enzymes in the liver tissues of control and the experimental groups of rats. It is clearly seen in Table 5, that citral had significantly restored the activity of all enzymes, which was close to the standard drug glibenclamide as compared to diabetic rats.

## Discussion

Diabetes is generally characterized by hyperglycemia resulting in deficiency or insensitivity to endogenous insulin with increased hepatic glucose production (42). In diabetes, apart from hyperglycemia, there is also disturbance in carbohydrate metabolic enzymes, which are responsible for the generation of ROS and leads to pathogenesis and progression of other diabetic problems (43). Therefore, managing antioxidant status and regulating glucose metabolic enzymes can be an effective way to control diabetes. As per our knowledge, this is the first *in vivo* study to evaluate the effect of Citral on oxidative stress and glucose metabolic enzymes in HFD/STZ induced diabetes in rats. In our study, diabetes and dyslipidemia were induced by a high-fat diet with low-dose of STZ injection in rats, which are prone to similar human diabetes (44). STZ destructs the beta cells of the pancreas in rats, which leads to insulin deficiency and feeding high-fat diet increases insulin resistance in rats (45). 

In this study, citral administration to diabetic dyslipidemic rats showed decreased glucose level in blood from high to normal level, which is good for the liver to get back its regular homeostasis. An explanation for the hypoglycemic action of Citral is either by lowering the level of blood glucose by increasing the glucose absorption from intestines or by regenerating beta cells to discharge more insulin. We have also seen the anti-hyperglycemic activity of Citral via oral glucose tolerance test (OGTT). In this study, it was found that glucose levels had increased during OGT testing in diabetic control rats and it remained high after 120 min (46). Although, the level of blood glucose in Citral treated diabetic rats reached a peak and after 120 min it came back to the normal level. This suggests that Citral improves tolerance of glucose and may enhance insulin discharge from existing b-cells. 

Diabetes induced by streptozotocin also causes a loss or degradation of structural proteins, resulting in severe body weight loss (47). In this study, we also found that the STZ-induced reduction in body weight was restored by Citral, as reported in earlier studies on photochemical or medicinal plant extracts in diabetic rats. (48).

 In diabetes, insulin deficiency and insulin insensitivity may affect the level of glucose and develop insulin resistance (49). In this study, it was seen that the insulin level in diabetic rats was decreased because the beta cells were either damaged or were not functioning properly. It was also observed that similar to glibenclamide-treated rats the insulin levels were increased in Citral-treated rats, confirming that citral can stimulate insulin secretion from beta cells or regenerate beta cells. Glycosylated hemoglobin (HbA1C) is considered a reliable marker for the diagnosis of diabetes. HbA1C was found to increase in diabetes mellitus in our study, and the amount of increase is directly proportional to the fasting blood glucose level (50). Citral and glibenclamide treatment of diabetic rats significantly decreased HbA1C when compared to normal control rats. The reason for this is that the level of sugar in the blood was regulated (51). 

 As we know that dyslipidemia is related with diabetes mellitus, and it has been confirmed that there are various types of disturbance in metabolic and regulatory processes happening during diabetes, due to which hyperlipidemic conditions occur in diabetic people (52). The plasma levels of FFAs, Pls, TC, LDL-C, and TG increase contributing to the diabetic dyslipidemic condition. In diabetes, insulin deficiency initially causes an increase in free fatty acid movement from adipose tissue. Increased level of free fatty acid can induce lipotoxicity in obesity and it has been revealed by many tissues like the pancreas, adipose, liver, etc. (53, 54). In our study, the level of lipid profile was also found to increase in streptozotocin induced diabetic dyslipidemic rats. Lipid profile levels were decreased after Citral administration in diabetic rats, this data supports the earlier findings reported on phytochemicals or medicinal plants. (55). The observed decline in plasma lipid profiles in Citral administered diabetic rats suggests that it is possibly due to the elevation of insulin level.

**Table 1:** Effect of Citral (30 mg/kg bw) on body weight

**Group**	**Body wt (initial) (gm)**	**Body wt (gm) after 15 days of treatment**	**Body wt (gm) after 30 days of treatment**
**Ctrl**	231±15.16	233±12.04	235±15.18
**HFD/STZ**	229±14.31#	146±9.61	151±12.09^#^
**HFD/STZ + Cit**	226±20.36*	170±7.96*	190±7.41*
**HFD/STZ + Glib**	224±19.24**	175±7.96**	200±11.18**
**HFD/STZ + Fen**	220±18.24**	158±13.05*	185±7.90**

Each value represents the mean±SE of six rats. Ctrl: Control rats; HFD/STZ: Diabetic rats; HFD/STZ + Cit: Diabetic rats treated with Citral; HFD/STZ + Glib: Diabetic rats treated with standard drug glybenclamide; HFD/STZ + Fen: Diabetic rats treated with standard drug fenofibrate.

# denotes significant difference compared with control rats;

* *P*<0.05;

** *P*<0.01;

*** *P*<0.001 denotes significant difference compared with diabetic control

**Table 2:** Effect of Citral (30 mg/kg bw) on oral glucose tolerance

**Group**	**Glucose (mg/dl) at different time intervals**				
	**0 min**	**30 min**	**60 min**	**90 min**	**120 min**
**Ctrl**	94±53.2	110±36.41	115±21.69	110±51.51	86±49.32
**HFD/STZ**	402±48.6	415±94.21	425±86.31^#^	435±86.95^#^	430±95.36
**HFD/STZ + Cit**	150±65.34	155±36.47	170±64.75	160±56.47**	145±86.14**
**HFD/STZ + Glib**	140±81.23	150±75.6	162±56.25	145±56.28**	130±88.24**
**HFD/STZ + Fen**	210±97.21	224±84.25	230±86.2	232±74.36	210±87.25

Each value represents the mean±SE of six rats. Ctrl: Control rats; HFD/STZ: Diabetic rats; HFD/STZ + Cit: Diabetic rats treated with Citral; HFD/STZ + Glib: Diabetic rats treated with standard drug glybenclamide; HFD/STZ + Fen: Diabetic rats treated with standard drug fenofibrate.

# denotes significant difference compared with control rats;

* *P*<0.05;

** *P*<0.01;

*** *P*<0.001 denotes significant difference compared with diabetic control

**Table 3:** Effect Citral (30 mg/kg bw) on blood glucose, HbA1C, and insulin

**Group**	**Blood glucose level** **(mg/dl)**	**Insulin (µU/ml)**	**Glycosylated hemoglobin (%Hb)**
**Ctrl**	110.6±26.96	17.54±5.42	4.2±2.74
**HFD/STZ**	320.8±81.66	6.45±3.68^#^	13.70±3.34^#^
**HFD/STZ + Cit**	143±26.59*	13.12±7.78*	7.19±2.83**
**HFD/STZ + Glib**	132±26.83***	16.10±4.06**	5.94±1.58**
**HFD/STZ + Fen**	206±59.41	9.92±4.01*	10.55±4.20

Each value represents the mean±SE of six rats. Ctrl: Control rats; HFD/STZ: Diabetic rats; HFD/STZ + Cit: Diabetic rats treated with Citral; HFD/STZ + Glib: Diabetic rats treated with standard drug glybenclamide; HFD/STZ + Fen: Diabetic rats treated with standard drug fenofibrate.

# denotes significant difference compared with control rats;

* *P*<0.05;

** *P*<0.01;

*** *P*<0.001 denotes significant difference compared with diabetic control

**Table-4:** Effect of Citral (30 mg/kg bw) on lipid profiles and free fatty acid.

**Group**	**TC(mg/dl)**	**TG(mg/dl)**	**FFAs (mg/dl)**	**PLs (mg/dl)**	**LDL-C (mg/dl)**
**Ctrl**	86±36.64	44.4±15.26	64±20.73	105±25.49	77±29.70
**HFD/STZ**	266±74.36^#^	104±51.28^#^	203±75.13^#^	216±59.41^#^	240±87.10^#^
**HFD/STZ + Cit**	136.06±25.01*	82.6±25.00	130.4±35.99*	128.6±32.01*	122.6±37.55
**HFD/STZ + Glib**	188.6±68.64*	81.6±22.14*	126.8±28.7*	168±88.84	120.75±86.14
**HFD/STZ + Fen**	126.8±51.22*	70.2±21.74**	106±25.59**	124.6±41.19*	106.2±56.50*

Each value represents the mean±SE of six rats. Ctrl: Control rats; HFD/STZ: Diabetic rats; HFD/STZ + Cit: Diabetic rats treated with Citral; HFD/STZ + Glib: Diabetic rats treated with standard drug glybenclamide; HFD/STZ + Fen: Diabetic rats treated with standard drug fenofibrate.

# denotes significant difference compared with control rats;

* *P*<0.05;

** *P*<0.01;

*** *P*<0.001 denotes significant difference compared with diabetic control

**Table 5:** Effect of Citral (30 mg/kg bw) on carbohydrate metabolism enzymes of the liver

**Group**	**Glucokinase (change in od x 10** ^3^ **/min/mg protein)**	**Hexokinase (µmol pi released/min/mg protein)**	**Glucose 6-phosphatase (µmol pi released/min/mg protein)**	**Pyruvate kinase (µmol pi released/min/mg protein)**	**Lactate dehydrgenase (n moles NADPH oxidized/ min/mg protein)**
**Ctrl**	2.51±1.01	2.17±0.81	9.31±1.25	42.31±2.63	3.24±0.89
**HFD/STZ**	1.89±0.89^#^	1.33±0.74^#^	21.21±3.69^#^	25.12±5.61^#^	5.21±1.07
**HFD/STZ + Cit**	2.21±0.58*	1.81±0.65*	12.21±1.04**	38.14±1.52*	3.84±1.05*
**HFD/STZ + Glib**	2.25±0.50**	1.89±0.56*	11.01±0.89**	39.61±1.02**	3.51±0.98*
**HFD/STZ + Fen**	2.05±1.12*	1.51±1.02*	12.09±1.63	28.12±2.36	4.21±1.34

Each value represents the mean±SE of six rats. Ctrl: Control rats; HFD/STZ: Diabetic rats; HFD/STZ + Cit: Diabetic rats treated with Citral; HFD/STZ + Glib: Diabetic rats treated with standard drug glybenclamide; HFD/STZ + Fen: Diabetic rats treated with standard drug fenofibrate.

# denotes significant difference compared with control rats;

* *P*<0.05;

** *P*<0.01;

*** *P*<0.001 denotes significant difference compared with diabetic control

The role and relationship of ROS in the development of diabetes has been well explained by many researchers. Hyperglycaemia generates free radicals, which leads to lipid peroxidation as well as membrane damage through which the formation of carbonyl groups after the protein oxidation occurs (56, 57). In the present study, the lipid peroxidation and activity of antioxidant enzymes in STZ/HFD induced diabetic dyslipidemic rats was researched. It was found that there has been a significant decrease in the activity of antioxidant enzymes, including SOD, CAT, GPX, and GR, with the reduction of GSH content in all tissues of diabetic rats, whereas the level of MDA has increased, which indicates oxidative stress in diabetes. As reported in earlier findings, various pathways of glucose metabolizing generate free radicals, which can be removed by phytochemicals. We have seen in our study that administration of Citral ameliorated the activity of SOD, Catalase, GPx, and GR and also increased GSH content in all tissues. Moreover, Citral also reduces the lipid peroxidation and protein carbonyl content in diabetic rats. Our oxidative stress results support the earlier research which states that phytochemicals are effectively treating tissue damage during diabetes (58, 59). Antioxidant effect of Citral may be due to its structure, chemically Citral is a mixture of terpenoids, which have the free radical removing property and can decrease the levels of reactive oxygen species (60). 

Glucose consumption (glycolysis) and production (gluconeogenesis) in diabetes are very important and both processes are taking place in the liver where several enzymes are involved. Insulin regulates carbohydrate metabolism via controlling the activities of numerous metabolic enzymes in the liver by modifying the uptake and consumption of glucose in target organs such as kidneys, skeletal muscles, and adipose tissues. Glucokinase and hexokinase are enzymes involved in the conversion of glucose to glucose-6 phosphate in the process of glycolysis (61, 62). Glucokinase is the susceptible marker of the glycolytic pathway in diabetes because it can increase the blood glucose consumption in the liver for glycogen storage (63).  Pyruvate kinase is involved in the last step of the glycolysis process. In our study, it was found that the activity of hexokinase, glucokinase and pyruvate kinase decreased in the liver of diabetic rats. Insulin deficiency is the characteristic of diabetes that leads to the impairment in the activity of these enzymes. Whereas the administration of Citral increased the activity of these enzymes due to which glycolysis can be activated and the use of glucose may increase. These results support the earliest finding of phytochemicals and medicinal plants (64–66). Hence the finding suggests that Citral was improving the glucose metabolism by increasing the utilization of glucose. Glucose-6-phosphatase is the key enzyme of gluconeogenic enzymes because it regulates both glycogenolysis and gluconeogenesis process of glucose metabolism (67, 68). The activity of glucose 6 phosphatase in diabetic rats was increased in the liver as compared to normal rats, probably due to insulin insufficiency because under normal conditions insulin acts to suppress gluconeogenic enzymes. Administration of Citral and glibenclamide inhibited the glucose 6 phosphatase enzyme activity as reported in other studies on phytochemicals (69), probably resulting in the restoration of blood glucose and glycogen content in the liver.

Lactate dehydrogenase (LDH) is the enzyme that helps to catalyze the interconversion of pyruvate to lactate and vice-versa in the glycolysis process (70). Increased level of LDH activity in diabetic rats as compared to normal control rats is linked with less insulin availability in diabetes (71). However, treatment of diabetic rats with Citral decreases the activity of LDH similar to glibenclamide treatment maybe because Citral controls the amount of pyruvate and NADH, thus it helps to process oxidation of glucose in mitochondria. Similar findings were reported by others (72, 73).

## Conclusion

The results of this experiment indicate that Citral controls the metabolism of carbohydrates in the liver of diabetic rats and restores the activity of regulatory enzymes that are involved in the production and consumption of glucose. It also protects and revives the pancreatic beta cells and helps to stimulate insulin. The data of our study also shows that Citral has an antioxidant property, maybe due to this, it possesses both anti-diabetics as well as antidyslipidemic properties. However, some more studies are necessary to understand the precise mechanism of antidiabetic as well as the antidyslipidemic activity of Citral.

## Conflicts of Interest

 All authors report that there are no conflicts of interest.
